# Violence, Misrecognition, and Place: Legal Envelopment and Colonial Governmentality in the Upper Skeena River, British Columbia, 1888

**DOI:** 10.1177/09646639231194450

**Published:** 2023-08-30

**Authors:** Matthew P. Unger

**Affiliations:** Department of Sociology and Anthropology, Concordia University, Montréal, Canada

**Keywords:** atmospheres, law, senses, colonialism, biopolitics, Foucault, legal atmospheres

## Abstract

This paper is concerned with exploring legal atmospheres during colonial expansionism and the early period of confederation of British Columbia. By describing the theatrical and performative aspects of legal colonialism, the archival documents from this time represent interesting, yet oft-overlooked, significances that attention to sensory and affective experiences captures. Examining “affective atmospheres” disclosed in such colonial settings reveals ways that the colonial regime promulgated its influence in non-rational, non-legal manners. As well, drawing out the material conditions of topography shows how the environment acts more than just a backdrop for the staging of legal expansionism, as it acts also as a constitutive force in the development of colonial legal arrangements. At the same time, the colonial regime was forgetful of these same contextual, topographical, and atmospheric origins of law insofar as it promulgated myths of the universality, objectivity, and superiority of English law.

## Introduction

Settled in wait on the upper Skeena River on the Northwest Coast of British Columbia a few kilometres from the Gitsxan communities of Gitanyow and Gitsegukla, the 74 militia men of Battery “C” cleared an area to set up camp, went hunting for big game, and made a trek up the nearby mountain. Commanded by the Provincial Secretary in 1888 to quell a feared Indigenous rebellion, the regimen was brought by military gunboat to the forks of the Skeena near Essington. Making an intimidating sight so far up the river, the military vessel eventually found its way up to Hazelton, a prominent trading town.^1^ However, apart from the displays of might and military strength, the regimen was never called on to intervene as the conflict was found to be resolved.

By describing the theatrical and performative aspects of legal colonialism, the archival documents from this time represent interesting, yet oft-overlooked, significances that attention to sensory and affective experiences seeks to capture (Appel, 2020). Indeed, examining the “affective atmospheres” (Anderson 2009, 2014) disclosed in such colonial settings reveals ways that the colonial regime promulgated its influence in non-rational manners. By non-rational, I mean factors that are typically thought of as exogenous to law itself as having significant influence on the development of law. For instance, by drawing out the material conditions of topography shows how the environment acts more than just a backdrop for the staging of legal expansionism, acting also as a constitutive force in the development of colonial legal arrangements. The colonial legal imaginary is comprised of the material, non-material, affective, topographical, and climatic conditions that lawmakers used to evoke atmospheres of awe and respect in Indigenous communities, Black communities, and communities of international migrants. Simultaneously, the colonial regime was forgetful of these same contextual, topographical, and atmospheric origins of law insofar as it promulgated myths of the universality, objectivity, and superiority of English law. In this way, I hope to show the aporetic nature of legal colonialism as it at once thought of itself as objective and necessary, but relied on contingent, theatrical, accidental, and ad hoc mechanisms for development.

In connecting current theories of biopolitics with that of atmospheres, this paper sets out to understand the exclusionary logics of forgetfulness and misrecognition embedded within the tactics of legal envelopment employed by the colonial regime in establishing sovereignty in British Columbia. I am interested in how English law became, in the terminology of Pierre Bourdieu (1977), “doxic” as it competed against other fields, knowledges, ways of life, and worldviews to the point that law became naturalized and its predominance established. Examining the situation on the Skeena River discloses the way early legal colonialism in British Columbia relied upon acts of misrecognition and violence to seize jurisdiction of land and law in competition with the dominion government and Indigenous communities. Additionally, this analysis helps show how legal colonialism mobilizes images of sovereignty to bolster local, contingent, and contextual forms of authority. By thinking through discourse in terms of legal envelopment and sovereignty, I set out to provide a fuller understanding of how atmospheres function by examining the colonial imaginary as it interacted with the topography and climate and generated experiences of legal performativity. To further examine how difference is an inherent part of legal atmospheres I turn to examining statements of affective bewilderment and exhortations for recognition by Indigenous chiefs in these asymmetrical encounters.

This paper, then, seeks to contribute to the already rich understanding of the various mechanisms, discourses, and inscriptions that comprise the colonial Canadian legal imaginary that sought to envelop the differing peoples, landscapes, and topographies of British Columbia into a single normative legal atmosphere. To explore legal envelopment through the perspective of atmospheres, I am looking to examine two areas of concern: one regarding the demonstrative (or) theatrical force that the colonial government employed with the intention to strike awe into the Indigenous populations; and the second regarding the non-rational, material, and non-material entities that came to influence the development of colonial law. In the end, I seek to de-reify legal atmospheres by describing the non-rational, theatrical, and constructed nature of atmospheres generated in legal contexts.

## Atmospheres

Studies in atmospheres emerged on the coattails of the affective turn as increasing attention was given to the ambiguous space of shared attention and mood (Griffero, 2016). The experience of an enveloping affective atmosphere as a palpable transpersonal or pre-personal experience has become increasingly theorized as having affective force within socio-political settings. Hermann Schmitz (2019) has contributed to a phenomenology of atmospheres wherein moods, emotions and affects that exist within spaces act as “half-things” that are significant yet intangible sources of affectivity. For Schmitz, “atmospheric feelings are not subjective-internal moods projected outside, but affective powers that exist discontinuously but objectively outside us and that authoritatively fill a given surfaceless (lived) space” (Griffero, 2016: 13–14). Gernot Böhme (1993, 2016), contributing to a philosophy of aesthetics and judgement, has suggested that the concept of atmospheres can expand our understanding of the aesthetic register of art and architecture as one way to document the often intangible but meaningful experiences of spaces. Aesthetics, for Böhme (2016), has shifted from a theory of judgement of primarily high artwork to the contemporary ubiquitous use and manufacture of atmospheres in almost all aesthetic products from advertising to art (48). Often drawing from Deleuze and Guattari's (1987)^1987^ Q3] musings on affect, theorists of atmospheres have pointed to shared understandings between the quotidian and the environmental uses of atmosphere wherein “intensities” of mood and affect take on the elemental qualities of air, climate, and topography (Philapopolous-Mihalopolous, 2013). Yet, in contrast to recent object-oriented materialisms, ambiguity, porousness, and openness to the environmental milieu characterize this approach, with authors such as Ben Anderson (2009, 2014) and Derek P. McCormack (2015, 2018) drawing out their more processual and posthuman aspects. Poignantly, Anderson (2009) writes that: “[a]ffective atmospheres are a class of experience that occur *before* and *alongside* the formation of subjectivity, *across* human and non-human materialities, and *in-between* subject/object distinctions” (78; emphasis original).

Drawing from these insights in studies in architecture and critical geography, authors have set out to understand the moods that constructed spaces and cities generate, focusing on the aesthetic register of these effects as one way to document the palpable and visceral, yet also intangible, ambiguous, and envelopmental, experiences of spaces. While early approaches to affect sought to establish its relational and social significance as a way of removing emotion solely from the purview of objectivist forms of psychological understandings, more recent interventions have challenged these interpretive frameworks by pointing to their conceptual difficulties. For instance, Bille and Simonsen (2019) offer a theory of atmospheres that he believes correct the deficits of recent contributions to affect theory that use the terminology of transmission and contagious flow between and through bodies to describe how affect circulate between actors in a milieu (cf. Brennan 2004, Massumi 2002, McCormack 2008). Instead, Bille and Simonsen (2019) suggest that to capture the mechanisms of affectivity that occur between bodies within a milieu, we must turn to phenomenology and practice theory to understand the ontological and affective connections between others in a milieu. In order to shift affect from a predominantly individualistic paradigm of the psychology of emotions, Bille and others explore atmospheres to understand how the porous subject comes to experience spaces in excess of the body.

Moreover, while earlier theoretical approaches tended to view atmospheres as complete or fully contained, more recent interventions have called attention to their incompleteness. An important intervention, made by McCormack (2018), is the notion of difference that pervades all experiences of atmosphere. In a processual and at times deconstructive understanding of atmospheres, McCormack challenges his readers to understand the unfinished and unclosed work of atmospheric objects and technologies, whereby difference is inherent within any experience of what he calls “elemental envelopment.” This difference takes the form of the unexpected within any enclosed atmospheric milieu, generating unpredictable effects and affects, varying experiences of intended objects, and a lack of clear boundaries between entities. McCormack (2018) writes that “envelopment is a process through which an atmospheric milieu is modified through a form of enclosure that generates different spheres of life that are inhabitable or uninhabitable to varying degrees” (29). From this perspective, envelopment is not merely a geographic term but something that can also help elucidate what it means to be amongst others in a socio-political milieu: “indeed, in many instances, life often depends upon the establishment and maintenance of an enveloping membrane that serves to separate and to some extent insulate an entity from the elemental conditions in which it is immersed” (McCormack, 2018: 29).

While McCormack eschews identity through much of his writing, this statement seems to naturalize the ontological necessity of defining identities in negativity. Or to say it differently, to understand entities through a privative logic of boundaries and identities could be a way of ontologizing imperialist Western ways of being. Following Peter Fitzpatrick (1992, 2001), we must remain diligent to continually excavate how Western liberal law embodies historically contingent, racist, and imperialist forms of privative logic, an idea that I explore further down. Our understanding of legal envelopment is enhanced by a focus on the ways that this privative logic, or logic of exclusion, is an integral aspect of legal predominance as it is established over great spaces and diverse peoples. However, as this project contends, attention to difference allows a greater understanding of ruptures and resistances which are an inherent aspect to this form of legal domination. To me, Foucault's (1984) description of heterotopias as spaces that encompass varying relations to time and space, power, and discourse helps expand conceptions of “lawscapes” (Philapopolous-Mihalopolous, 2013, 2014) as contoured by difference as much as exclusionary biopolitical logics. He writes:

The space in which we live, which draws us out of ourselves, in which the erosion of our lives, our time and our history occurs, the space that claws and gnaws at us, is also, in itself, a heterogeneous space. In other words, we do not live in a kind of void, inside of which we could place individuals and things. We do not live inside a void that could be colored with diverse shades of light, we live inside a set of relations that delineates sites which are irreducible to one another and absolutely not superimposable on one another. (1984: 3)

While the ontology of legal colonialism is privative and depends on exclusion through inclusion, we can understand atmospheric envelopment, and by extension envelopment through legal atmospheres, as a form of heterotopia. Legal atmospheres are heterotopic in that they encompass sets of relations, experiences, and ideas, in which difference, ruptures, and resistances are intrinsic to a particular atmospheric milieu. By de-reifying the legal ontology of legal colonialism and the envelopment it sought atmospheres, then, becomes a way to examine how entities within a particular milieu are all a part of that milieu but not reducible to the milieu itself. This irreducibility also signifies how these entities are never fully present to that same milieu but are both affected by and affect it. So, it allows an account of how peoples, entities, discourses, and experiences are never autonomous and singular, but porous and unfolding. It is the permeable and unfinished experiences of entities that allow accidental and unforeseen events, experiences, and affects within legal situations that seek envelopment.

## Legal Envelopment and the Myth of Sovereignty

This sense of envelopment has much in common with Philapopolous-Mihalopolous (2013, 2014) rich description of lawscapes as an all-encompassing affective and spatial experience of law in the city. He suggests that law is ubiquitous in today's world, touching every aspect of one's lived experience in modern late-capitalist society. The “hyperesthesia” that is a product of complex city life is governed by law to its very core, whereby law overwhelms, touches, regulates, and recedes from every felt sensory experience within the city (Howes, 2005). Philapopolous-Mihalopolous (2013) describes the open ecology of the city as functioning in an integrative yet dissimulative manner with the open normativity of the law, whereby they reinforce each other's necessity yet still maintain a relative autonomy.

Understanding law as something that is integrally related to spatial arrangements has been theorized by authors such as David Delaney (2001, 2010), Nicholas Blomley (2003, 2004), and Peter Sloterdijk (2006). These contributions help us understand how legal envelopment shapes the everyday world and quotidian experience of self and others. For instance, Delaney (2001) argues that including space in legal analysis allows an understanding of how spatio-legal arrangements have direct and material effects in the world. He writes: “*Law matters*, at least because these metaphysical distinctions are realized—perhaps “concretized” is the better term—on segments of the material world” (Delaney, 2001: 498; emphasis original). In other words, the legal imaginary is comprised of informal understandings of space and place that directly impact the recognition of people, places, and things when transcribed into law. More recently, Philapopolous-Mihalopolous (2014) has signalled a critique of theories relating to law and space by suggesting that “they are characterised by a compartmentalisation of the non-human in relation to a spatially determined human community” in which “the human remains a central figure of perception, performance or action” (86). In other words, the task in theorizing the relationship between law and space, according to Philapopolous-Mihalopolous, is to challenge the Western, human-centric epistemological and ontological foundations of law itself. What theories of atmospheres add to the spatial interpretation of law is not only a historical (or genealogical) approach towards law's emergence, but also a description of how law has been contoured by the material conditions of space, by human and non-human relations, absences, resistances, misrecognitions, plurality, accidents, technicity, violence, forgetfulness, contradictions, and aporias.

While providing a theory to understand the openness of spaces and entities within legally intensive milieus, attending to atmospheres, as Sumartojo and Pink (2018) put forth, also forces us to think through the ways that our experiences are contoured by spatiality, temporality, and mobility. By this, they mean that, while atmospheric analyses examine how spaces are engineered in particular ways, this does not preclude a diversity of experience of these spaces, how time is disclosed and experienced, and how one moves through these porous fields. It is the interaction between manufactured spaces and the diversity of experiences that gives expression to a politics of atmospheres, but also provides the capacity to understand ruptures, resistances, and difference within, for example, legally intensive spatial arrangements. So, while these spaces—whether architectural, public, or legal—are often designed with the intention of a particular affect and experience, how people experience them cannot be absolutely predicted. Understanding the standpoints that people bring to these spaces is important for understanding how these atmospheres can never be total or totalizing. Similarly, the symbolic register of design and manufacturing processes comes out of a particular intentionality and discursive milieu that need to be incorporated in an analysis of atmospheres. Indeed, Sumartojo and Pink (2019), quoting Ranciere (2004), draw out the aesthetic quality of politics as “a delimitation of spaces and times, of the visible and invisible, of speech and noise, that simultaneously determines the place and stakes of politics as a form of experience” (22). So then, these atmospheric arrangements and assemblages and their discursive intentionality draw out the capacities of sensing and knowing a particular space. Put another way, atmospheres disclose a directivity in attention that can only be experienced differentially by those that are part of the milieu. In a legally intensive experience, this directivity has implications for what is contoured by law or singled out as something that constitutes wrongdoing.

In a colonial legal context such as that of British Columbia in the mid to late 1800s, manufactured legal atmospheres ran up against long established normative and legal worlds with varying degrees of acceptance and resistance. What has been shown by scholars such as Pavlich (2017), Pavlich and Unger (2016), Foster (1984, 1994), and Loo (1994, 1995) is that these collisions had the effect of creating new individualist accusatory apparatuses that contrasted sharply with the normative worlds of Indigenous understandings of wrongdoing. This draws us towards what Sumartojo and Pink (2019) have described as the temporality, or the “eventfulness” of atmospheres (24). Affective and temporal dimensions are significant characteristics of legal atmospheres, as we see in the communications between settlers, lawmakers, and the colonial offices. The troubled relations between settler communities and the Indigenous communities on the Northwest coast were contoured by previous historical encounters and initial contact, contingent moments of misrecognition and violence, and future anticipation. Thus, normative and affective atmospheres were impactful in terms of how law was carried out and even contributed to the promulgation of English legal envelopment, as the fear of the absences within the Western liberal legal form served to threaten law itself.

The eventfulness of legal atmospheres can also be understood through theatrical and demonstrative events, through which lawmakers intended to evoke an atmosphere of awe and respect in Indigenous communities so that they were more susceptible to the impressive power and rationality of European law and civilization. However, the intended atmospheres of these displays of might depended not only on technology and military prowess, but also on discursive conditions that privileged these modes of engagement, which many people enclosed within these atmospheres did not share. One way that we can understand these discursive conditions that lay at the foundation of colonial legal atmospheres is through Peter Fitzpatrick's (1996, 2001) examination of the mythology of Western law and its contradictions. In these texts, Fitzpatrick examines the Western interpretation of myth since the Enlightenment—including early anthropological classificatory schemas of primitiveness, irrationality, religion, and myth—to understand how law fulfils the same conditions of that which it has been defined against. What I mean is that the attempt to create a rational, objective, and universal law depends on a host of assumptions and presuppositions that can be characterized as mythological in themselves. Taking inspiration from Derrida's (1974) deconstructive logic, Fitzpatrick (1996) argues that replete through Western law's development are a series of irresolvable contradictions and aporias implicit within the attempt to universalize a culturally contingent normative and legal horizon, which are “reconciled” by the mythology that led to its development. These contradictions can be traced through Western philosophy, from the Enlightenment to contemporary legal positivism, being part of the attempt to form autonomous knowledge in the same manner as physics, psychology, and other new knowledges. Early legal knowledges and legal positivisms circumvented the social ground of law in the understanding of law as an autonomous “thing” that is bounded and self-originary. In other words, the Enlightenment legal project worked towards the evisceration of the cultural content of the universal legal framework. As such, at the foundation of the Western liberal legal framework is the erasure of its own mythological origins.

The dissimulative nature of the colonial legal lawscape is thus expressed in the production of prefigurative atmospheres of forgetfulness and misrecognition. Manufactured legal spaces depend on these mythical grounds to create legal atmospheres through culturally specific rituals to evince their objectivity and self-perceived superiority. These effaced performances are especially—violently and vehemently—present in colonial legal engagements that mean to envelop Indigenous populations with differing cultural, normative, and legal horizons.

The “open ecology” within legal atmospherics promulgates the potentiality of law even as law reached its own limits. Legal colonialism functioned as a process of atmospheric envelopment that sought to make universal a particular way of seeing the world and understanding wrongdoing to strong normative effect. The drawing of attention to aspects of the world not previously “seen” is shown in archival documents where settler society members communicated their fears of Indigenous uprisings with lawmakers in Victoria, BC's capital. As well, new categories of wrongdoing and their criminalization were shown in the diversity of experiences and understandings of legal colonialism within the Indigenous communities. At the same time this collision was characterized by asymmetrical relations as the totalizing “open ecology” of English law met, often violently, with that of Indigenous legal pluralisms.

## Atmospheres and the Skeena River Event of 1888

There are several ways of reading colonial legal expansionism through an atmospheric analysis. This can be explored in the archival narratives that recount experiential and affective descriptions of the environment during policing, investigations, manhunts, and circuit court travels; sovereignty politics that depend on theatrical displays of force; the accidents, and haphazard and ad hoc policing that recursively led to further legal expansionism; and the misunderstandings and misrecognitions that derive from particular affective experiences of law. In order to explore legal atmospheres as a heterotopic space that is characterized by difference, resistance, and ruptures, I turn now to the Skeena River event of 1888. This is a complicated and multivalent series of incidents and interactions that still resonates within today's Indigenous–Canadian government relationship as the most recent Gitsxan and Wet'suwet'sen blockades and protests in 2019 and 2020 over the construction for the Coastal Link LNG pipeline have shown (McCreary, 2013; Barker, 2015).^2^ In its examination of archival documents less well-explored by historians of this event, this section is concerned with looking into how the colonial regime usurped territory and legal normative worlds through accidental and performative ways. In fact, military engagement with the material, non-material, and non-human became a metonymy for the war-like engagement with Indigenous communities during this time of legal colonialism. Furthermore, in the collision of normative and spatio-legal horizons, affective atmospheres related to territory and land claims can be read as a critical component that shaped the direction of differing points of conflict of this and prior encounters.

I am relying on several histories for this brief description. However, as Williams (2000) and others have mentioned, these histories also relied on documentary evidence given through colonial archival documents, which have already been subject to translation and transcription by colonial lawmakers to make them legible to the dominion at that time. While Marius Barbeau (1973) extensive ethnography traces the experiences of those who lived through the event or were given a part in history through oral tradition, those experiences have still been translated and transcribed by the anthropologist. Moreover, as Williams (2000) and Cassidy (1983) have pointed to, the asymmetry in the relationship between the government and Indigenous peoples would have impacted the reliability of the translation of Indigenous peoples’ representations of themselves in legal situations. Many difficulties of translation make it challenging to tell the full story of what happened during this event, yet a brief description of the event is in order.

The winter after the devastating epidemic on the Northwest Coast of British Columbia, the family of a prominent chief named Kamalmuk (or Jim) of the village of Kitwankul on the Upper Skeena River, now known as Gitanyow, had lost two sons to the disease. According to ethnography in the first half of the twentieth century, some of the inner tensions between neighbouring communities led the mother, wife of Kamalmuk, to blame the Shaman, Neatsqua, of the neighbouring Gitsegukla, for the deaths of her sons. Seeking recompense for the death of their sons, Kamalmuk shot and killed the Shaman (Barbeau, 1973, Johnson 1977, Cassidy, 1983).^3^ Recognizing that he had made a grievous mistake that had angered the Gitsegukla community, Kamalmuk sought to make amends and resolve the difficulties in their traditional ways. The community refused to accept the compensation and further retribution against Kamalmuk became a possibility, after which Kamalmuk went into hiding. To stave off further bloodshed, and encouraged by the bishop of the local mission, the chiefs of Gitsegukla recalled a written exhortation from an officer aboard a naval gunship in 1872 that urged the Gitxsan communities to never take the law into their own hands and declared that any conflict needed to be resolved using English law (Williams, 2000; Cassidy, 1983). The chiefs and the bishop then counselled the community to allow English law to resolve this conflict. In response to the request to resolve the conflict between Gitanyow and Gitsegukla, the provincial government tasked Constable Greene to search for and arrest Kamalmuk and take him to Port Simpson for trial. After much delay, the legal party, including Indian Agent Charles Todd, travelled upriver to resolve this and another conflict that had emerged in the meanwhile. After the manhunt, three constables caught up to Kamalmuk, a scene that eventually ended up with Constable Greene shooting Kamalmuk fatally in the back. The death was unsanctioned by the communities, who then hardened against English law as they became determined to settle the conflict according to Gitxsan tradition. The communities advised the constable to leave the area with their conditions of compensation for the killing of one of their community members.

As legal historians of British Columbia have noted, the fraught experience of contact and competition for land and resources lay as the broader context of the conflict shaping atmospheres of territory beginning with the Omineca gold rush of 1870–71. One of the initial points of conflict was an event in 1872 when a Gitxsan village had been overrun by miners, which led to a fire that swept through the town. In response, the Gitxsan blockaded the Skeena River to protest the destruction wrought by the influx of traders and miners in their territories. The event was eventually resolved according to Gitxsan customary procedures of conflict resolution, as were some of later conflicts that occurred through the 1880s. Another significant contextual aspect of legal colonial encroachment is the experience of epidemics of communicable diseases such as measles, which washed through the Tsimshian communities in the winter of 1887–88, during which time it was reported that over 200 children had died (Johnson, 1984). This and the colonial government's attempt to secure land and resources in the 1880s prompted further distrust, difficulties, protests, and tensions between settlers and Indigenous groups, all of which lay as the affective atmospheric background to the Skeena River events.

The legal intervention during the set of events of 1888 appears to have had deleterious effects in settler—Indigenous relations in the area, as the miscommunications and accidents set off a panic regarding the potential for Indigenous reprisals against the settler communities. These miscommunications between constables, missionaries, and provincial and dominion lawmakers prompted aggressive military style “gunboat diplomacy” (Gough, 1984). Negotiations in Victoria amongst the provincial government led to the dispatch of a naval gunship, along with regimen “Battery C” of 84 local militia, joined by a dozen constables and superintendent H.B. Roycroft, to quell the feared rebellion. Once at Hazelton, the captain of the mission, J.G. Holmes, received word that the troubles in the communities had calmed and that the battery would wait at Essington until the services of the militia were called for. Having settled on a more permanent ground for the duration, the constables and militiamen set forth making camp, hunting, and surveying the land. Yet, the performative aspect of their presence was a tangential, but significant part of the colonial regime's tactic to prevent Indigenous resistance. The captain described the effect of the presence of the militia on the local communities and virtual enemies, human or nonhuman.^4^ Recounting the “building huts and clearing a small parade ground,” Holmes reported how the group faced topographical bewilderment as rifle practice was “rather difficult owing to the impassable nature of the country.” He was comforted by this fact as he felt their enemies would have a difficult time penetrating such forest: “we were quite safe from flank attack, provided the flanks of the river were carefully searched for a reasonable distance back, no force could travel through this country.” More descriptions of the physical feats of the group coincided with images of heroic masculinity:

Pending receipt of word from Mr Roycroft, all the Officers and many of the N.C. Officers and men took advantage of our situation to climb the adjacent mountains, most of them 4000 to 6000 feet high and snow-capped. This was work of the hardest description and showed the pluck and endurance of all who undertook it in a marked way. Considerable game such as bears, mountain goats, etc. were killed and the skins brought back as trophies.^5^

Even though no military engagement was required, the officer recounts a virtual war against the land and non-human animals in which the conquering of the “wilderness” acted as a metonym for the legal encounter with Indigenous communities. This is confirmed when Holmes writes: “I mention this as showing how adaptable the men were to work which was very similar to what we should have had to perform, had the expedition proceeded to fight its way to Hazelton.” However, as the letter to the Adjutant General of Militia in Ottawa continues, it appears that things were not as they seemed. For despite the nearly 100 men at their disposal, “had there been any serious fighting we were too weak a force for the work.” Holmes continues, with an air of disappointment:

Although the expedition has had a peaceful termination, I feel and am happy to know, that it will have a permanent salutary effect on the Indian element in the North West. Many Indians from all parts of the country were assembled at the mouth of the river for the salmon fishing and the presence of a ship of war so far up the river, and an armed force remaining independently of her, which has been carried out for the first time in this country will have the effect of widely disseminating a knowledge of power of the government to put down promptly all future uprisings.^6^

For Holmes, the mere presence of the warship was to shape the imaginations of the communities in an area that had little contact with the colonial regime up till this point. Establishing legal envelopment, in this instance, meant quite simply that the law, reinforced by military might, could reach areas that the regime deemed to be remote. Military might, for the British Columbia regime equalled legal sovereignty and the display of the of the warship was an affective display of this kind of legal colonialism.

Additionally, after the large group of constables and military officers saw the conflict resolved according to Gitxsan customary law, the colonial legal framework interpreted the event in such a way that prompted the lead constable to threaten the communities to adopt English law once again. To impose upon the communities English law's power and force, Police Superintendent Roycroft sought to demonstrate impartial English legal procedures by trying the constable who had shot Chief Kamalmuk in the back as he was fleeing. As a further show of force, they also held a series of demonstrations of military might and attempted to incorporate the chiefs within the colonial legal framework by making them special constables. Yet, the success of Gitxsan customary law and the failure of English law to resolve the difficulties only served to recursively reinforce (for lawmakers at the time) the necessity of the colonial legal imaginary, even as it flamed further territorial disputes, resentment, tensions, and distrust. In other words, the “open ecology” of the law was evoked even as it sowed further disorder and violence in the relationships between the different groups involved in the conflict.

While the constabulary activity and military intervention in the Upper Skeena River to “resolve” this and prior conflicts in the area had further deleterious effects on settler-Indigenous relations, the events only served to increase calls to adopt English law. In their communications with the chiefs of the Upper Skeena, the provincial government made it clear that colonial categories of wrong-doing and criminalization were to be adopted. In a provincial commission that was held between 1887 and 1888 after Indigenous communities requested to be heard regarding land claims rights, the state of English law in their communities, and general community relations, the provincial secretary wrote to the chiefs: “It is by the Queen's law that all the people, Indians and whites alike, are now governed, and those who disobey that law must be punished, no matter what they may have been accustomed to before. Besides,” he continued, “the Queen's law is better than yours, as you will see.”^7^ Not all the chiefs interviewed at these commissions agreed with such a sentiment. Take the response from the chief of Kincolith, Matthew Naas, which reads as conciliatory, pragmatic, and plural, but also shows bewilderment at the perceived arrogance of the colonial encroachment:

We want sufficient land now for our numbers. We want food, salmon, berries, animals for food and furs, timber for houses, canoes and boxes, bark for mats. Now these things are got in different places, and we want land where we can get them. The land where we get fruit does not yield timber; we go to different places for different things…We did not ask the Government to come and touch our land. They came and when they commenced, then we began to see what we want and what we don’t want.

If I’m reading this statement correctly, the chief is saying that the engagement with the colonial regime forced an evaluation of the meaningful capacities of the land for the Indigenous communities. This seems to also imply that they expected to be respected in their evaluations for sharing the land with the colonists. The statement “we began to see what we want and what we don’t want” suggests that the encounter with the settlers and the colonial government shaped how the communities saw their own land, in turn shaping territorial atmospheres. He continues:

And now, if the good chiefs and our chiefs put their hands to a paper, that paper will stay with us and with our children, and children's children, and all will be peace, and that is what we want…If we make a mistake now, we are making it not only for ourselves but for our children, who will suffer. Our children and their children will honour anything done by the chiefs present here to-day. My father was a chief of Metlakatlah, and my mother a Naas woman; that is why I am on the Naas. My father did not want his land and his father's land called a reserve; that is why he got up and left the country for Alaska; and we don’t like the word “reserve” any more than he did; but now, if we have a treaty, we will be willing to live on a reserve.^8^

Here, the chief is outlining the gravity of the agreement in sharing land—that this one decision will have effects for generations, and they want to be sure that they are making the right decision for their community and the generations after. While this chief is exhorting the colonial government for formal recognition of their use of land, this quote also shows how Indigenous conceptions of legal plurality reflect differing experiences of temporality and space as the chief speaks about their deep connection to the land. David Mackay, a chief of Greenville, responds with a similar level of bewilderment at the differing normative worlds connecting time and space, and shaping relations to land:

What we don’t like about the Government is their saying this: “We will give you this much of land.” How can they give it when it is our own? We cannot understand it. They have never bought it from us or our forefathers. They have never fought and conquered our people and taken the land in that way, and yet they say now that they will give us so much land—our own land. These chiefs do not talk foolishly, they know the land is their own; our forefathers for generations and generations past had their land here all around us; chiefs have had their own hunting grounds, their salmon streams, and places where they got their berries; it has always been so. It is not only during the last four or five years that we have seen the land; we have always seen and owned it; it is no new thing; it has been ours for generations.^9^

And finally, Am-Clamman, a sub-chief of Kit-wil-luk shilts responds with:

You saw us laughing yesterday when Neis Puck got up and spoke, because you opened the book and told us the land was the Queen's and not the Indians’. That is what we laughed at. No one ever does that, claiming property that belongs to other people. We nearly fainted when we heard that this land was claimed by the Queen. The land is like the money in our pockets, no one has a right to claim it. We all agree with what David said, that we should be paid for our land outside of what we want for ourselves.^10^

With this statement, we can almost get the palpable sense of their affective experience of bewilderment and absurdity as a result from the colonial legal declaration of territory and ownership. The bewilderment in these quotes appear to arise from what the Indigenous communities have perceived as a violence of misrecognition and betrayal, as well as a violation of the legal plurality that arises from agreements and treaties about boundaries, ownership, and belonging (cf Anker 2021, Borrows 2005). That is, the difference that the Indigenous communities seem to be advocating was misrecognized by the colonial regime as a threat that needed to be incorporated within English Law. What we see, then, is an almost ontological misrecognition of the “form” of law whereby the plurality and principles of agreement advocated by the chiefs were not legible to the Western legal order, prompting aggressive legal colonial performances.

## Envelopmental Legal Atmospheres

As we have understood through the media and news sources about the recent Gitsxan and Wet'suwet’en protests, blockades, and conflict with police over the LNG pipeline, the conflict between the Indigenous communities on the Skeena and the colonial regime is not arising out of nowhere, but out of a long colonial history of misrecognition, violence, maltreatment, and the lack of treaties with the Tsimshian, Nisga’a, Gitxsan, and Wet'suwet’en Indigenous peoples. The Skeena River event from 1888 is just one example of legal colonial expansionism in which conflict between settler society and Indigenous communities set forth legal machinations in ad hoc manners as missionaries, constables, and other settler arrangements attempted to instil a particular legal imaginary to the exclusion of all other forms of community relations, customary laws, and forms of conflict resolution. It is clear from the archival documents, ethnography, and writing about the event that legal colonialism did not bring law and order as the colonialists thought, but rather, as Fitzpatrick (2001) also mentions, incredible disorder and chaos (71). If atmospheres, as Anderson (2014) has suggested, are ambiguous spaces that envelope with differences, the 1888 confrontation between Indigenous communities and the colonial legal regime discloses several ways in which legally intensive spatial arrangements have palpable, indelible effects. These legal atmospheres, however, are far from coherent and consonant with everyone involved and affected, and we see in the archival documents, at least, profound differences, resistances, and ruptures.

Not only does an atmospheric analysis from the early confederation period in British Columbia allow us to examine the affective interaction of space, normativity, and legal envelopment but it also allows us a window into the non-rational components that lawmakers used to extend law's reach further into Indigenous communities and territories. Finally, by examining beyond just what the legal regime recounted, by drawing on and allowing marginalized voices that were not legible to the legal regime. What we observe with the Skeena River event is the virtual staging of war with the land, topography, human and non-human actors, and normative horizons when human conflict was not amenable to receiving “resolution” through colonial legal mechanisms. This suggests that the openness of the legal form is also contoured not only by the discursive, mythological, prefigurative parameters I’ve examined, but also by the material and affective conditions that lawmakers, settlers, and Indigenous communities experienced. So, while the communities themselves resolved their conflicts according to their customary law, the accidental and haphazard mobilization of law only served to further the “open ecology” of the legal form. BC's colonial legal context shows that violent mis(or non)recognition of others are a core component of its form confirming Foucault's (2003) insistence on the racist foundations of Western law. And as such, legal atmospheres are always constituted by withdrawal, difference, and a presencing that never quite arrives because the entities that are enclosed are never fully present to experience the legal haphazardly mobilized atmospheres.

## Notes

1. British Columbia, Legislative Assembly, “PAPERS Relating to the Commission Appointed to Enquire into the State and Condition of the Indians of the North-West Coast of British Columbia” (L. BC Sessional Papers. Victoria, BC: Government Printer, 1888), http://dx.doi.org/10.14288/1.0062087.

2. See, for example, “Wet'suwet’en chiefs, blockades and coastal gaslink: a guide to the dispute over a B.C. pipeline,” *The Globe and Mail*, 14 January 2020, https://www.theglobeandmail.com/canada/british-columbia/article-wetsuweten-coastal-gaslink-pipeline-rcmp-explainer/.

3. Johnson, I. V. B. (VII-C-36M), “The Skeena uprising 1888” [1977], Box: 259, f22, Canadian Museum of History.

4. Skeena River Indian troubles - Col. Sec'y [Colonial Secretary], 1888/07/23, no. 194, asks for particulars resp. [respecting]. Privy Council Minutes, 26 November–6 December 1888 (R.G. 2, Series 1, Volume 407), Library and Archives Canada, http://central.bac-lac.gc.ca/.redirect?app=ordincou&id=45104&lang=eng.

5. Skeena River Indian troubles - Col. Sec'y [Colonial Secretary], 1888/07/23, no. 194, asks for particulars resp. [respecting]. Privy Council Minutes, 26 November–6 December 1888 (R.G. 2, Series 1, Volume 407), Library and Archives Canada, http://central.bac-lac.gc.ca/.redirect?app=ordincou&id=45104&lang=eng.

6. Skeena River Indian troubles - Col. Sec'y [Colonial Secretary], 1888/07/23, no. 194, asks for particulars resp. [respecting]. Privy Council Minutes, 26 November–6 December 1888 (R.G. 2, Series 1, Volume 407), http://central.bac-lac.gc.ca/.redirect?app=ordincou&id=45104&lang=eng.

7. British Columbia, Legislative Assembly, “PAPERS Relating to the Commission Appointed to Enquire into the State and Condition of the Indians of the North-West Coast of British Columbia” (L. BC Sessional Papers, Victoria, BC: Government Printer, 1888), http://dx.doi.org/10.14288/1.0062087.

8. British Columbia, Legislative Assembly, “PAPERS Relating to the Commission Appointed to Enquire into the State and Condition of the Indians of the North-West Coast of British Columbia” (L. BC Sessional Papers, Victoria, BC: Government Printer, 1888), http://dx.doi.org/10.14288/1.0062087.

9. British Columbia, Legislative Assembly, “PAPERS Relating to the Commission Appointed to Enquire into the State and Condition of the Indians of the North-West Coast of British Columbia” (L. BC Sessional Papers, Victoria, BC: Government Printer, 1888), http://dx.doi.org/10.14288/1.0062087.

10. British Columbia, Legislative Assembly, “PAPERS Relating to the Commission Appointed to Enquire into the State and Condition of the Indians of the North-West Coast of British Columbia” (L. BC Sessional Papers, Victoria, BC: Government Printer, 1888), http://dx.doi.org/10.14288/1.0062087.
